# Silenced lncRNA SNHG14 restrains the biological behaviors of bladder cancer cells via regulating microRNA-211-3p/ESM1 axis

**DOI:** 10.1186/s12935-020-01717-7

**Published:** 2021-01-22

**Authors:** Rui Feng, Zhongxing Li, Xing Wang, Guangcheng Ge, Yuejun Jia, Dan Wu, Yali Ji, Chenghao Wang

**Affiliations:** Department of Urology, Zhenjiang Hospital of Chinese Traditional And Western Medicine, 18 Tuanshan Road, Zhenjiang, Jiangsu 212002 China

**Keywords:** Bladder cancer, Long noncoding RNA small nucleolar RNA host gene 14, MicroRNA-211-3p, Endothelial cell-specific molecule 1, Proliferation, Migration, Invasion, Apoptosis

## Abstract

**Background:**

Bladder cancer (BCa) is a malignant tumor that occurs on the mucosa of the bladder, in which dysregulated long non-coding RNAs (lncRNAs) are involved. This study investigated the effect of lncRNA small nucleolar RNA host gene 1 (SNHG14) on the biological characteristics of BCa cells from microRNA (miR)-211-3p/ESM1 signaling axis.

**Methods:**

BCa tissues and the matched normal tissues were collected to test SNHG14, miR-211-3p and ESM1 levels. SNHG14, miR-211-3p and ESM1 levels in BCa cell lines (T24, 5637, UMUC-3 and EJ) and normal bladder epithelial cells SV-HVC-1 were detected for screening the cell lines for follow-up experiments. T24 and UMUC-3 cells were transfected with different plasmids of SNHG14, miR-211-3p or ESM1 to observe the biological characteristics of BCa cells by MTT, colony formation, Transwell assays and flow cytometry. Tumor xenograft was implemented to inspect tumor growth in vivo. The targeting relationships of SNHG14, miR-211-3p and ESM1 were verified by bioinformatics software, RNA pull down assay and luciferase reporter assay.

**Results:**

Enhanced SNHG14, ESM1 and suppressed miR-211-3p were found in BCa tissues and cells. SNHG14 up-regulated ESM1 via competitive binding with miR-211-3p. Decreased SNHG14 or up-regulated miR-211-3p depressed cell cycle entry, colony formation, invasion, migration and proliferation abilities, and facilitated apoptosis of BCa cells. Decreased SNHG14 or up-regulated miR-211-3p reduced the tumor volume and weight of nude mice with BCa, as well as promoted apoptosis and restrained proliferation of tumor cells. miR-211-3p inhibition or ESM1 overexpression reversed the effects of down-regulation of SNHG14 on BCa, and miR-211-3p up-regulation or ESM1 downregulation reversed the effect of SNHG14 overexpression on BCa. SNHG14 targeted miR-211-3p to regulate ESM1 expression.

**Conclusion:**

Our study highlights that silenced SNHG14 or elevated miR-211-3p represses the tumorigenic ability of BCa cells, which may be linked to ESM1 knockdown.

## Background

Bladder cancer (BCa), the most general malignant solid tumor in the urogenital tract, is the 4th most familiar cancer in males and 7th most usual solid tumor in females [1]. BCa takes over 3% of cancer diagnoses globally [2], and the morbidity and mortality of males are four times that of females [3]. There are a lot of risk factors, consisting of tobacco use, family history, occupational exposure, long-term urinary catheter use, and Schistosoma haematobium infection [4]. Nowadays, the treatments for BCa mainly include surgery, radiotherapy and chemotherapy [5]. About 75% of the lesions show up around the bladder at diagnosis, while 25% are examined in the regional lymph nodes or faraway locations [4]. Despite various therapies for BCa, the clinical outcome is unsatisfied because of metastasis, recurrence and drug resistance [6]. With the poor prognosis, an in-depth understanding of BCa will pave a new way for exploring new therapeutic strategies.

Long noncoding RNA (lncRNA) is classified as a form of noncoding RNA transcript [7]. LncRNA small nucleolar RNA host gene 14 (SNHG14) is oncogenic as to cell invasion, migration, proliferation, and chemo-resistance in various malignancies [8]. SNHG14 is the one that participates in BCa development [9]. Additionally, it is reported that SNHG14 can promote the occurrence and development of BCa by targeting miRNA [9]. MicroRNAs (miRNAs) are endogenous ~ 22 nucleotide noncoding RNAs that can modulate protein-coding genes by binding to complementary sites in the 3′-untranslated region (3′UTR) of their targets [10]. Some studies have shown the connection of miRNAs with BCa progression, such as miR-212-3p and miR‑125b‑5p [1, 11]. There are studies showing that miR-211-3p plays a role in colorectal cancer, ovarian cancer and pancreatic cancer [12–14]. Moreover, miR-211-3p has been proven to be down-regulated in gastric cancer [15] and lung adenocarcinoma [16], thus it is assumed to inhibit BCa as well. Furthermore, by predicting the binding site of SNHG14 and miR-211-3p, we found that SNHG14 had a potential binding site with miR-211-3p. Endothelial cell-specific molecule 1 (ESM1) or endocan is a gene taking a part in the angiogenic activation that acts during hypoxia-induced retinal neovascularization in animal models [17, 18]. ESM1 expression has also been referred in stromal endothelial cells of human tumors besides hepatocellular carcinoma [19]. Besides, ESM1 is found to be highly expressed in blood vessels in aggressive BCa tissues [20]. However, how ESM1 is involved in the development of BCa remains unclear. In addition, we predicted the binding site of miR-211-3p and ESM1 through the bioinformatics website. Thus, we proposed the scientific hypothesis that SNHG14 may affect the occurrence and development of BCa by modulating miR-211-3p/ESM1 axis. For verifying our assumption, we conducted this study to explore the effect of SNHG14/miR-211-3p/ESM1 axis on the biological characteristics of BCa cells.

## Materials and methods

### Compliance with ethical standards

The study was approved by the Institutional Review Board of Zhenjiang Hospital of Chinese Traditional And Western Medicine and followed the tenets of the *Declaration of Helsinki*. All participants signed the document of informed consents. The protocol was approved by the Institutional Animal Care and Use Committee of Zhenjiang Hospital of Chinese Traditional And Western Medicine and met the ethical requirements of animal experiments.

### Study subjects

From January 2015 to December 2017, 62 cases of cancer tissues from BCa patients (the tumor group) and their paired adjacent normal tissues (> 5 cm from the cancerous tissue, the normal group) were collected from the urosurgery department of Zhenjiang Hospital of Chinese Traditional And Western Medicine after radical cystectomy. Among them, 50 patients were male, 12 were female, 44 aged > 50 years, and 18 aged ≤ 50 years. All patients had complete clinical medical record. The BCa patients were included if they received total cystectomy but not chemotherapy or radiotherapy before surgery or any other treatments that might affect the operation of this study; sufficient bladder tissue samples could be obtained for the experiment without affecting the conventional clinicopathological diagnosis. Clinicopathological diagnosis confirmed that these patients were BCa (mainly urothelial carcinoma), rather than bladder benign tumors or other types of tumors. The World Health Organization grading method in 2004 was used for pathological grading. Clinical staging was identified by the tumor node metastasis (TNM) clinical staging system of Union for International Cancer Control and American Joint Committee on Cancer in 2009. Patients were followed up in outpatient clinics or by telephone. The follow-up lasted for 30 months and ended in June 2019.

### Cell culture, grouping and transfection

Normal urothelial cells SV-HUC-1, BCa cell lines T24, 5637, UMUC-3 and EJ (American Type Culture Collection, VA, USA) were seeded in RPMI-1640 medium. The cells were passaged according to 1: 5 and incubated for 5–7 d. The cells (80% confluence) with vigorous proliferation and good growth state were selected for the experiment. SNHG14 and miR-211-3p levels in each cell line were determined by reverse transcription quantitative polymerase chain reaction (RT-qPCR), and the cell lines with the highest and lowest relative expression levels were chosen for subsequent cell experiments.

T24 cells were grouped into the blank (no transfection); the si-negative control (NC) (transfected with silenced SNHG14 plasmid NC); the si-SNHG14 (transfected with silenced SNHG14 plasmid); the mimic NC (transfected with miR-211-3p mimic NC); the miR-211-3p mimic (transfected with miR-211-3p mimic); the si-SNHG14 + inhibitor NC (transfected with silenced SNHG14 plasmid and miR-211-3p inhibitor NC); the si-SNHG14 + miR-211-3p inhibitor (transfected with silenced SNHG14 plasmid and miR-211-3p inhibitor); the si-SNHG14 + oe-NC (transfected with silenced SNHG14 plasmid and oe-ESM1 NC); the si-SNHG14 + oe-ESM1 (transfected with silenced SNHG14 plasmid and oe-ESM1) groups.

UMUC-3 cells were assigned into the blank (no transfection); the overexpression (oe)-NC (transfected with overexpressed SNHG14 plasmid NC); the oe-SNHG14 (transfected with overexpressed SNHG14 plasmid); the inhibitor NC (transfected with miR-211-3p inhibitor NC); the miR-211-3p inhibitor (transfected with miR-211-3p inhibitor); the oe-SNHG14 + mimic NC (transfected with elevated SNHG14 plasmid and miR-211-3p mimic NC); the oe-SNHG14 + miR-211-3p mimic (transfected with overexpressed SNHG14 plasmid and miR-211-3p mimic); the oe-SNHG14 + sh-NC (transfected with overexpressed SNHG14 plasmid and sh-ESM1 NC); the oe-SNHG14 + sh-ESM1 group (transfected with overexpressed SNHG14 plasmid and sh-ESM1) groups.

Si-NC, si-SNHG14, oe-NC, oe-SNHG14, oe-ESM1, sh-NC and sh-ESM1 were purchased from Sangon Biotechnology Co. Ltd. (Shanghai, China) while mimic NC, miR-211-3p mimic, inhibitor NC and miR-211-3p inhibitor from GenePharma Co. Ltd. (Shanghai, China).

The cells were seeded on 12-well plates containing penicillin/streptomycin-free complete culture solution (1.5 mL/well) for 24 h before transfection. BCa cell lines T24 and UMUC-3 with 60% confluence were transiently transfected via lipofectamine 2000 (Invitrogen, Carlsbad, California, USA). Then, at 6 h post transfection, the medium was changed, and cells were cultured for 48 h for follow-up experiments.

### RT-qPCR

Total RNA in tissues and cells was extracted with Trizol kit (Invitrogen), and the concentration and purity were determined by DU-800 nucleic acid spectrophotometer. Glyceraldehyde-3-phosphate dehydrogenase (GAPDH) and U6 were applied as loading controls. PCR primers were designed and synthesized by Takara (Dalian, China) (Table 1). In compliance with the instructions of RNA reverse transcription kit (Takara), RNA was reversely transcribed into cDNA for PCR amplification. The data were analyzed via 2^−ΔΔCt^ method.

**Table 1:** PCR primer sequence

	Primer sequence (5’-3’)
SNHG14	Forward: 5’-GGGTGTTTACGTAGACCAGAACC-3’
	Reverse: 5’-CTTCCAAAAGCCTTCTGCCTTAG-3’
miR-211-3p	Forward: 5’-GTCGTATCCAGTGCGTGTCGTGG-3’
	Reverse: 5’-AGTCGGCAATTGCACTGGATACG-3’
U6	Forward: 5’-TTAGCATGGCCCCTGC-3’
	Reverse: 5’-TGCGTGTCGTGGAGTC-3’
ESM1	Forward: 5’-AGCTGGAATTCCATGAAGAG-3’
	Reverse: 5’-TCTCTCAGAAGCTTAGCCG-3’
GAPDH	Forward: 5’-GAGTCAACGGATTTGGTCGT-3’
	Reverse: 5’-GATCTCGCTCCTGGAAGATG-3’

Note: SNHG14, long noncoding RNA small nucleolar RNA host gene 14; miR-211-3p, microRNA-211-3p; ESM1, endothelial cell-specific molecule 1; GAPDH, glyceraldehyde-3-phosphate dehydrogenase

### Western blot analysis

BCa tissues and adjacent normal tissues in liquid nitrogen were ground to powder. BCa cells were added with protein lysis solution (Radio-Immunoprecipitation assay: Phenylmethylsulfonyl fluoride was 100: 1) and centrifuged at 20,000 rpm to take the supernatant. The 10% sodium dodecyl sulfate separation gel and spacer gel were prepared. The sample was mixed with the buffer solution and boiled at 100 °C for 5 min. After ice bath and centrifugation, the sample was processed by electrophoresis separation and transferred to the nitrocellulose membrane. The membrane was blocked with 5% skim milk powder, incubated with primary antibodies ESM1 (1: 200, Santa Cruz Biotechnology, Inc, Santa Cruz, CA, USA) and GAPDH (1: 1000, Cell Signaling Technology, Beverly, MA, USA), and with the secondary antibody, horseradish peroxidase-labeled IgG (1: 1000, Wuhan Boster Biological Technology Co., Ltd., Hubei, China). The membrane was immersed in enhanced chemiluminescence solution (Pierce, Rockford, IL, USA) and developed via LAS4000 mini chemiluminescence imager. The gray value was measured via the imaging system software and normalized to GAPDH. Protein imprinting images were analyzed with ImageJ2x software.

### Dual luciferase reporter gene assay

The binding sites of SNHG14 and miR-211-3p were predicted via bioinformatics software Diana tools. SNHG14 wild type (WT) and mutant type (MUT) amplification primers were designed. Restriction endonuclease Hind III and Mlu I were applied for synchronous enzyme digestion of pMIR-Report carrier plasmid and target gene. T4 ligase was employed to transform enzyme-digested target genes and pMIR-Report plasmid into Escherichia coli JM109 strains. Colony PCR and recombinant plasmid were treated with enzyme digestion and finally sequenced. MiR-211-3p mimic and mimic NC (GenePharma) were co-transfected to T24 and UMUC-3 cells with SNHG14-WT and SNHG14-MUT respectively. Luciferase activity in lysed cells was detected via the luciferase detection kit (Promega, Madison, WI, USA) and an ultraviolet spectrophotometer (Bio-Rad, Inc., Hercules, CA, USA).

Bioinformatics software https://cm.jefferson.edu/ was used to predict the binding region of miR-211-3p and ESM1 mRNA 3′UTR. ESM1 3′UTR containing the binding site of miR-211-3p was synthesized via PCR method and the ESM1 3′UTR WT and ESM1 3′UTR MUT plasmids were constructed. T24 and UMUC-3 cells were co-transfected with miR-211-3p mimic or mimic NC with ESM1-3′UTR-WT or ESM1-3′UTR-MUT. Transfected for 48 h, firefly luciferase activity and renilla luciferase activity in cells were determined with a fluorescence detector and a dual luciferase reporter gene assay kit (Promega).

### RNA pull-down assay

Bio-miR-211-3p-WT, Bio-miR-211-3p-MUT (sequence mutation complementary to SNHG14) and Bio-NC (a random miRNA not complementary to SNHG14) were designed and synthesized by GenePharma. After T24 and UMUC-3 cells grew to 80% – 90% confluence, the three miRNAs were transfected for 48 h. Then the cells were lysed and the protein lysates were incubated with M-280 streptavidin coated with magnetic beads (Sigma-Aldrich Chemical Company, St Louis, MO, USA), and then the magnetic beads were washed with buffer. Finally, the adsorbed protein-nucleic acid complex on magnetic beads was eluted. Total RNA was extracted via Trizol, and SNHG14 was detected by RT-qPCR.

### 3-(4, 5-dimethylthiazol-2-yl)-2, 5-diphenyltetrazolium bromide (MTT) assay

T24 and UMUC-3 cell suspensions were seeded into 96-well plates at 5 × 10^4^ cells/per well, with 6 parallel wells. The cells (80% confluence) were treated according to the above experimental groups, and then cultured for 24, 48, 72 h and 96 h respectively and incubated with 20 μL 5 mg/mL MTT solution (Sigma). Then each well was added with 150 μL dimethyl sulfoxide (Sigma) and the optical density (OD_490 nm_) value was detected on a microplate reader.

### Colony formation assay

After T24 and UMUC-3 cells were detached with trypsin, 200 cells were cultivated into 6-well plates for 2–3 weeks. The culture was terminated once visible cell colonies were formed. The cells were fixed with 4% paraformaldehyde for 30 min, stained with Giemsa solution for 60 min, and the number of cell colonies was counted under the microscope.

### Transwell assay

T24 and UMUC-3 cells were detached. Each Transwell chamber was coated with 80 μL matrigel (1: 8, Becton, Dickinson and Company, Franklin lake, New Jersey, USA), and matrigel was not added in migration assay. Then, 1 × 10^2^ cells with 100 μL serum-free Dulbecco’s Modified Eagle Medium were incubated in the upper chamber for 24 h (lower chamber containing complete medium). Then a cotton swab was applied to wipe off cells in the upper chamber, and the cells were fixed with 4% paraformaldehyde for 15 min and stained with crystal violet for 10 min. Cell images were taken in five fields under the microscope, and the number of cells penetrating the membrane was counted.

### Flow cytometry

Cell cycle: Detached T24 and UMUC-3 cells were suspended and centrifuged. Then the cells were re-suspended in PBS to reach 1 × 10^6^ cells/mL and prepared into a single cell suspension, which was centrifuged at 2000 rpm. The cells were fixed with 500 μL 70% cold ethanol, centrifuged at 2000 rpm for 3 min and water-bathed with 100 μL RNase A. After that, cells were stained with 400 μL propidium iodide (PI) and detected on a flow cytometer (the red fluorescence at 488 nm was recorded).

Apoptosis: after detachment, T24 and UMUC-3 cells were centrifuged and made into cell suspensions with PBS. The cells (200 μL, 1 × 10^6^ cells/mL) were centrifuged, re-suspended in 100 μL binding buffer, and reacted with Annexin-V-FITC (2 μL, 20 μg/mL). Then the cells, along with 300 μL PBS and 1 μL PI (50 μg/mL) were detected in a flow cytometer within 30 min. Annexin-V was used as the horizontal axis and PI as the vertical axis. The upper left quadrant showed mechanically damaged cells, the upper right quadrant showed late apoptotic cells or necrotic cells; the lower left quadrant showed negative normal cells; and the lower right quadrant showed early apoptotic cells.

### Tumor xenograft in nude mice

In this study, 42 female BALB/c-nu nude mice (16–24 g, 4 weeks old) from Experimental Animal Center of Jiangsu University (Jiangsu, China) were kept in the aseptic laminar flow room of the animal laboratory of Zhenjiang Hospital of Chinese Traditional And Western Medicine at 24–26 °C with 45%–50% humidity. The pad, feed, feeding cage and drinking water bottle were sterilized by high pressure and replaced twice a week. The mice injected with T24 cells were grouped into the blank, si-NC, si-SNHG14, mimic NC, miR-211-3p mimic, si-SNHG14 + inhibitor NC, and si-SNHG14 + miR-211-3p inhibitor groups. In UMUC-3 cells, there were the blank, oe-NC, oe-SNHG14, inhibitor NC, miR-211-3p inhibitor, oe-SNHG14 + mimic NC, and oe-SNHG14 + miR-211-3p mimic groups (n = 3). The transfected T24 and UMUC-3 cells (3 × 10^6^ cells/200 μL) were subcutaneously injected into the neck and back under aseptic conditions. The maximum length (L) of the tumor was measured weekly, and the width (W) perpendicular to the maximum L was measured. The tumor volume was calculated (V = 1/2 × L × W^2^). At 6 weeks post injection, the nude mice were euthanized, and the subcutaneous tumors were isolated, weighed and photographed. Tumor formation rate = subcutaneous tumor formation rate/total number of mice × 100%. With the infection time as the X-axis and tumor volume as the Y-axis, and the xenograft tumor growth curve was formed.

### Transferase-mediated deoxyuridine triphosphate-biotin nick end labeling (TUNEL) staining

Tumor tissues from mice were fixed in 4% paraformaldehyde and embedded in paraffin. Then, 5 pieces of tissues (5-mm) were deparaffinized, reacted with 1% protease K solution (50 μL) and inactivated in 0.3% H_2_O_2_. After that, the sections were added with TUNEL solution, developed by 50 μL Converter-POD and 2% diaminobenzidine (DBA). Then, the sections were counter-stained with hematoxylin and treated with gradient ethanol (50, 70, 90, 100%) and xylene. Sealed with neutral gum, the sections were observed under a optical microscope in 10 fields. The cells with brown nucleus were apoptotic cells, and those with blue nucleus were normal cells. The ratio of the number of brown cells/blue cells represented cell apoptosis rate.

### Immunohistochemistry

The paraffin sections of mouse tumor tissues were deparaffinized, directly immersed in 3% H_2_O_2_, blocked with 10% normal goat serum and probed with rabbit anti Ki67 polyclonal antibody (ab15580, 1:200, Abcam). Also, the sections were reacted with biotin-labeled goat anti-rabbit IgG secondary antibody (1:1000, ab6721, Abcam), added with SP solution and developed by DBA. Treated with hematoxylin and mounted with neutral gum, the sections were observed by a microscope (Olympus, Japan). The cytoplasm stained into yellowish-brown was defined as positive staining. Under a light microscope, 5 high-powered fields were randomly selected. The positive rate was the ratio of positive cells/total cells.

### Statistical analysis

SPSS 21.0 (IBM Corp. Armonk, NY, USA) statistical software was applied to analyze the data. The measurement data were expressed as mean ± standard deviation. The t test was used in the comparison between the two groups, one-way analysis of variance (ANOVA) in that among multiple groups, and Tukey’s post hoc test in pairwise comparisons. The enumeration data were represented as rate or percentage, and Chi square test was used for comparative analysis. *P* was a two-sided test, and predictors were kept if they were significant at a *P* value of 0.05 or smaller.

## Results

### Enhanced SNHG14, ESM1 and suppressed miR-211-3p are found in BCa tissues

**Fig. 1:**
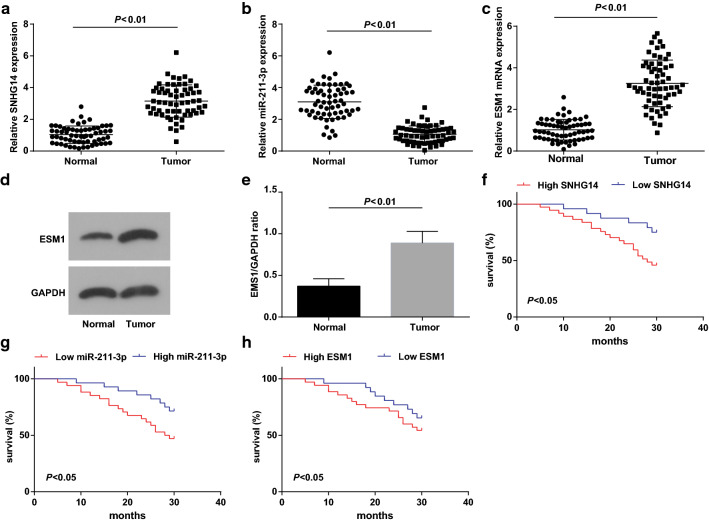
Elevated SNHG14, ESM1 and decreased miR-211-3p are found in BCa tissues. **a** The expression of SNHG14 in bladder tissues of the two groups detected via RT-qPCR; **b** The expression of miR-211-3p in bladder tissues of the two groups detected via RT-qPCR; **c** The expression of ESM1 mRNA in bladder tissues of the two groups detected via RT-qPCR; **d** Protein bands of ESM1 in bladder tissues of the two groups detected via western blot analysis; **e** The expression of ESM1 protein in bladder tissues of the two groups detected via western blot analysis. **f** The effect of SNHG14 expression on the prognosis of BCa patients; **g** The effect of miR-211-3p expression on the prognosis of BCa patients; **h** The effect of ESM1 expression on the prognosis of BCa patients; n = 62; * vs the normal group, *P* < 0.05; The measurement data were expressed as mean ± standard deviation. The data were compared via using t test in two groups

RT-qPCR and western blot analysis showed that SNHG14 (Fig. 1a) and ESM1 (Fig. 1c–e) levels in the tumor group were apparently increased, and miR-211-3p level (Fig. 1b) was obviously decreased in contrast with the normal group (all *P* < 0.05). Collectively, elevated SNHG14 and ESM1 and inhibited miR-211-3p were presented in BCa.

### SNHG14 in BCa is connected with TNM stage, tumor invasion stage and lymph node metastasis

SNHG14 expression was apparently higher in BCa tissues in contrast with adjacent normal tissues, and there were 37 cases with high expression and 25 cases with low expression. miR-211-3p expression was lower in BCa tissues than that of adjacent normal tissues. High miR-211-3p expression was detected in 28 cases, and low miR-211-3p expression in 34 cases. ESM1 expression was raised in BCa tissues versus adjacent normal tissues. A total of 35 cases demonstrated high ESM1 expression and 27 cases displayed low ESM1 expressions. It was suggested that SNHG14, miR-211-3p and ESM1 in BCa were linked with TNM stage, tumor invasion stage and lymph node metastasis, but was not with gender, age, pathological grade or tumor size (Tables 2, 3 and 4). Based on the median value of SNHG14, miR-211-3p or ESM1 expression, BCa patients were allocated into low expression group and high expression group. Kaplan–Meier analysis was carried out to invesitigate the effects of SNHG14, miR-211-3p and ESM1 expression levels on the survival and prognosis of BCa patients. The findings revealed that patients with high SNHG14 or ESM1 expression had worse prognosis. In addition, patients with high miR-211-3p expression had better prognosis (Fig. 1f–j).

**Table 2:** The expression of lncRNA SNHG14 in BCa is related to the patients’ TNM stage, tumor invasion stage and lymph node metastasis

Clinicopathological data	Case	LncRNA SNHG14 expression		*P*
		High expression group (n = 37)	Low expression group (n = 25)	
Age				1.000
50 or less	18	11	7	
> 50	44	26	18	
Gender				0.198
Male	50	32	18	
Female	12	5	7	
Tumor size (cm)				0.288
< 3.5	40	26	14	
3.5 or higher	22	11	11	
Tumor invasion stage (T)				< 0.001
Tis/Ta/T1	34	13	21	
T2/T3 or above	28	24	4	
TNM staging				< 0.001
0/I	33	10	23	
II/III/IV	29	27	2	
Lymph node metastasis (N)				0.002
N0	47	23	24	
N1 or more	15	14	1	
Pathological grade				0.605
Low	28	18	10	
High	34	19	15

**Table 3:** The expression of miR-211-3p in BCa is related to the patients’ TNM stage, tumor invasion stage and lymph node metastasis

Clinicopathological data	Case	miR-211-3p expression		*P*
		High expression group (n = 28)	Low expression group (n = 34)	
Age				0.272
50 or less	18	6	12	
> 50	44	22	22	
Gender				0.117
Male	50	20	30	
Female	12	8	4	
Tumor size (cm)				0.118
< 3.5	40	15	25	
3.5 or higher	22	13	9	
Tumor invasion stage (T)				< 0.001
Tis/Ta/T1	34	22	12	
T2/T3 or above	28	6	22	
TNM staging				0.002
0/I	33	21	12	
II/III/IV	29	7	22	
Lymph node metastasis (N)				0.006
N0	47	26	21	
N1 or more	15	2	13	
Pathological grade				0.450
Low	28	11	17	
High	34	17	17

**Table 4:** The expression of ESM1 in BCa is related to the patients’ TNM stage, tumor invasion stage and lymph node metastasis

Clinicopathological data	Case	ESM1 expression		*P*
		High expression group (n = 35)	Low expression group (n = 27)	
Age				0.400
50 or less	18	12	6	
> 50	44	23	21	
Gender				0.748
Male	50	29	21	
Female	12	6	6	
Tumor size (cm)				0.108
< 3.5	40	26	14	
3.5 or higher	22	9	13	
Tumor invasion stage (T)				< 0.0001
Tis/Ta/T1	34	12	23	
T2/T3 or above	28	24	4	
TNM staging				0.002
0/I	33	12	21	
II/III/IV	29	23	7	
Lymph node metastasis (N)				0.008
N0	47	22	25	
N1 or more	15	13	2	
Pathological grade				0.798
Low	28	15	13	
High	34	20	14	

### SNHG14 and ESM1 are up-regulated while miR-211-3p is down-regulated in BCa cell lines

**Fig. 2:**
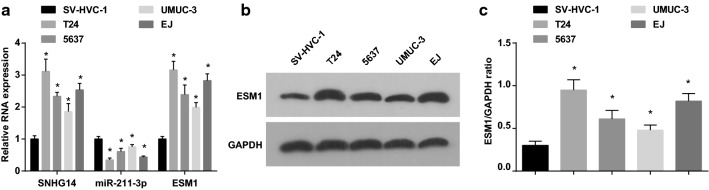
SNHG14 and ESM1 are up-regulated while miR-211-3p is down-regulated in BCa cell lines. **a** The expression of SNHG14, miR-211-3p and ESM1 in T24, 5637, UMUC-3, EJ and SV-HVC-1 cell lines tested via RT-qPCR; **b** Protein bands of ESM1 in T24, 5637, UMUC-3, EJ and SV-HVC-1 cell lines tested via western blot analysis; **c** The expression of ESM1 protein in T24, 5637, UMUC-3, EJ and SV-HVC-1 cell lines tested via western blot analysis. * vs the SV-HVC-1 cell lines, *P* < 0.05. The measurement data were expressed as mean ± standard deviation. One-way ANOVA was functioned for comparison among multiple groups, and Tukey’s post hoc test was used for pairwise comparison

SNHG14, miR-211-3p and ESM1 levels in BCa cell lines (T24, 5637, UMUC-3 and EJ) and normal bladder epithelial cells SV-HCV-1 were examined by RT-qPCR and western blot analysis. It was showed that SNHG14 and ESM1 levels in T24, 5637, UMUC-3 and EJ cell lines were apparently elevated, and miR-211-3p level was clearly decreased in contrast with SV-HCV-1 cells (all *P* < 0.05). In BCa cell lines, SNHG14 and ESM1 levels were the highest and miR-211-3p was the lowest in T24 cells, and UMUC-3 cells showed opposite expression levels (Fig. 2a–c). Therefore, T24 cells were selected for the follow-up SNHG14 inhibition and miR-211-3p overexpression cell experiment, and UMUC-3 cells for SNHG14 overexpression and miR-211-3p inhibition cell experiment.

### SNHG14 up-regulates ESM1 via competitively combining with miR-211-3p

**Fig. 3:**
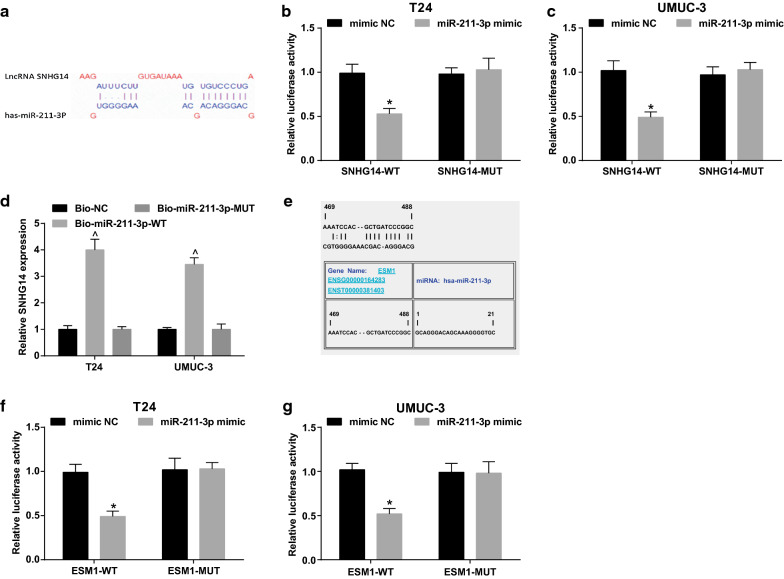
There is a binding relationship between SNHG14 and miR-211-3p and a targeting relationship between miR-211-3p and ESM1. **a** The binding sites of SNHG14 and miR-211-3p predicted via Diana tools; **b** The binding relationship between SNHG14 and miR-211-3p in T24 cells verified via dual luciferase reporter gene assay; **c** The binding relationship between SNHG14 and miR-211-3p in UMUC-3 cells verified via dual luciferase reporter gene assay. **d** The binding relationship between SNHG14 and miR-211-3p verified via RNA pull down assay. **e** The binding site of miR-211-3p and ESM1 predicted by Jefferson; **f** The binding relationship between miR-211-3p and ESM1 in T24 cells verified via dual luciferase reporter gene assay; **g** The binding relationship between miR-211-3p and ESM1 in UMUC-3 cells verified via dual luciferase reporter gene assay; * vs the mimic NC group, *P* < 0.05; ^ vs the Bio-NC group, *P* < 0.05; The measurement data were expressed as mean ± standard deviation. The data were compared via using t test in two groups. One-way ANOVA was functioned for comparison among multiple groups, and Tukey’s post hoc test was used for pairwise comparison

Online analysis software predicted a specific binding region between SNHG14 and miR-211-3p (Fig. 3a). Dual luciferase reporter gene assay indicated that in contrast with the mimic NC group, luciferase activity of WT SNHG14 + miR-211-3p mimic group was distinctly decreased (*P* < 0.05), indicating that there was a binding relationship between SNHG14 and miR-211-3p (Fig. 3b, c).

In RNA-pull down assay, in contrast with the Bio-NC group, SNHG14 level in T24 and UMUC-3 cells was obviously increased in the Bio-miR-211-3p-WT group (both *P* < 0.05). It indicated that Bio-miR-211-3p-WT could promote the enrichment of SNHG14, which confirmed that SNHG14 could reduce the dissociation degree of miR-211-3p in BCa cells by binding to miR-211-3p (Fig. 3d).

The online bioinformatics software predicted the targeting relationship between miR-211-3p and ESM1 and discovered the targeting site between the two (Fig. 3e). T24 and UMUC-3 cells were co-transfected with ESM1-WT plasmid and miR-211-3p mimic, and the results showed that luciferase activity of the ESM1-WT + miR-211-3p mimic group was obviously decreased in comparison with the ESM1-WT + mimic NC group (*P* < 0.01) (Fig. 3f–g). It was delineated that SNHG14 competitively bound to miR-211-3p to elevate ESM1 expression.

### Decreased SNHG14 increases miR-211-3p expression and decreases ESM1 expression; elevated miR-211-3p decreases ESM1 expression in BCa cells

**Fig. 4:**
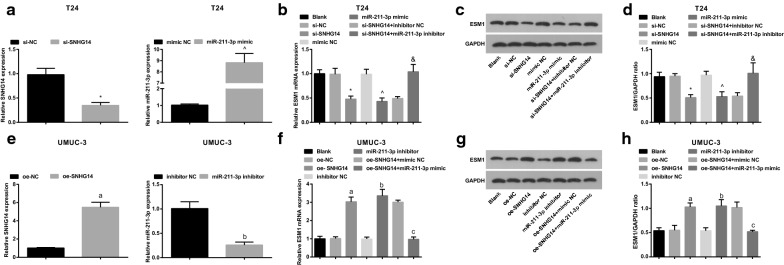
Silenced SNHG14 increases miR-211-3p expression and decreases ESM1 expression; elevated miR-211-3p decreases ESM1 expression in BCa cells. **a** The efficiency of SNHG14 and miR-211-3p in T24 cells was verified by RT-qPCR; **b** ESM1 mRNA expression in T24 cells detected via RT-qPCR; **c** Protein bands of ESM1 in T24 cells tested via western blot analysis; **d** The expression of ESM1 protein in T24 cells detected via western blot analysis; **e** The efficiency of SNHG14 and miR-211-3p in UMUC-3 cells was verified by RT-qPCR; **f** ESM1 mRNA expression in UMUC-3 cells detected via RT-qPCR; **g** Protein bands of ESM1 in UMUC-3 cells tested via western blot analysis; **h** The expression of ESM1 protein in UMUC-3 cells detected via western blot analysis. * vs the si-NC group, *P* < 0.05; ^ vs the mimic NC group, *P* < 0.05; and vs the si-SNHG14 + inhibitor NC group, *P* < 0.05; a vs the oe-NC group, *P* < 0.05; b vs the inhibitor NC group, *P* < 0.05; c vs the oe-SNHG14 + mimic NC group, *P* < 0.05. The experiment was repeated independently for 3 times, and the measurement data were expressed as mean ± standard deviation. One-way ANOVA was functioned for comparison among multiple groups, and Tukey’s post hoc test was used for pairwise comparison

The transfection efficiency of SNHG14 and miR-211-3p was verified by RT-qPCR to conduct subsequent experiments (Fig. 4a, e). RT-qPCR and western blot analysis showed that in T24 cells, versus the si-NC group, ESM1 level in the si-SNHG14 group was obviously decreased (*P* < 0.05). With the mimic NC group by contrast, ESM1 expression in the miR-211-3p mimic group was obviously decreased (*P* < 0.05). In comparison with the si-SNHG14 + inhibitor NC group, ESM1 expression in the si-SNHG14 + miR-211-3p inhibitor group was obviously elevated (*P *< 0.05) (Fig. 4b–d). Versus the si-SNHG14 + oe-NC group, ESM1 level was increased in the si-SNHG14 + oe-ESM1 group (Additional file 1: Fig. S1a, b). miR-211-3p inhibition or ESM1 overexpression reversed the effect of SNHG14 down-regulation on BCa.

In UMUC-3 cells, with respect to the oe-NC group, ESM1 level in the oe-SNHG14 group was obviously increased (*P* < 0.05). With the inhibitor NC group by contrast, ESM1 expression in the miR-211-3p inhibitor group was obviously elevated (*P* < 0.05). In comparison with the oe-SNHG14 + mimic NC group, ESM1 expression in the oe-SNHG14 + miR-211-3p mimic group was obviously reduced (*P *< 0.05) (Fig. 4f–h). Versus the oe-SNHG14 + sh-NC group, reduced ESM1 was displayed in the oe-SNHG14 + sh-ESM1 group (Additional file 1: Fig. S1c, d), indicating miR-211-3p up-regulation or ESM1 down-regulation reversed the effect of SNHG14 overexpression on BCa.

### Decreased SNHG14 or up-regulated miR-211-3p inhibits proliferation and colony formation ability of BCa cells

**Fig. 5:**
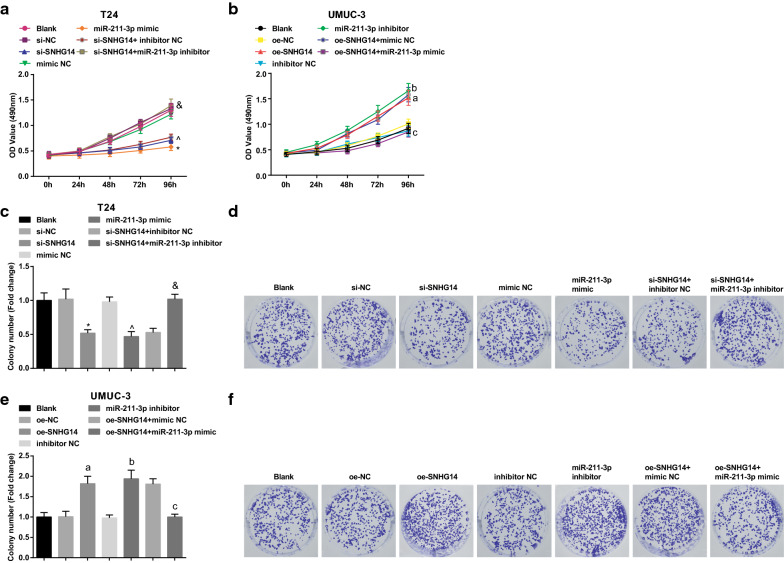
Silenced SNHG14 or up-regulated miR-211-3p strains proliferation and colony formation ability of BCa cells. **a** The proliferation of T24 cells after transfection tested via MTT assay; **b** The proliferation of UMUC-3 cells after transfection tested via MTT assay; **c** The number of colonies of T24 cells after transfection detected via colony formation assay; **d** The colony formation ability of T24 cells in each group detected via colony formation assay; **e** The number of colonies of UMUC-3 cells after transfection detected via colony formation assay; **f** The colony formation ability of UMUC-3 cells after transfection detected via colony formation assay; * vs the si-NC group, *P* < 0.05; ^ vs the mimic NC group, *P* < 0.05; & vs the si-SNHG14 + inhibitor NC group, *P* < 0.05; a vs the oe-NC group, *P* < 0.05; b vs the inhibitor NC group, *P* < 0.05; c vs the oe-SNHG14 + mimic NC group, *P* < 0.05. The experiment was repeated independently for 3 times, and the measurement data were expressed as mean ± standard deviation. One-way ANOVA was functioned for comparison among multiple groups, and Tukey’s post hoc test was used for pairwise comparison

Cell proliferation is one of the important physiological functions of living cells, an important characteristic of organisms, and the basis of organism growth, development, reproduction and heredity. Tumor cell proliferation activity is closely related to its growth, invasion, recurrence, metastasis and prognosis. In the present research, the changes of cell proliferation ability and cell colony formation ability after transfection were detected via MTT and colony formation assays. The results manifested that in T24 cells, versus the si-NC and the mimic NC groups, the proliferation ability and the number of cell colonies in the si-SNHG14 and the miR-211-3p mimic groups were obviously decreased (all *P* < 0.05). With respect to the si-SNHG14 + inhibitor NC group, cell proliferation and colony formation ability were enhanced in the si-SNHG14 + miR-211-3p inhibitor group (both *P *< 0.05) (Fig. 5a, c, d). By contrast with the si-SNHG14 + oe-NC group, the cell proliferation was enhanced in the si-SNHG14 + oe-ESM1 group (Additional file 1: Fig. S1e, f).

In UMUC-3 cells, with the oe-NC and the inhibitor NC groups by contrast, the proliferation ability and the number of cell colonies were obviously increased in the oe-SNHG14 and the miR-211-3p inhibitor groups (all *P* < 0.05). Versus the oe-SNHG14 + mimic NC group, the proliferation ability and the number of cell colonies in the oe-SNHG14 + miR-211-3p mimic group were obviously reduced (both *P *< 0.05) (Fig. 5b, e, f). With respect to the oe-SNHG14 + sh-NC group, the oe-SNHG14 + sh-ESM1 group characterized by suppressed cell proliferation (Additional file 1: Fig. 1g, h). It was indicated that the cells with down-regulated SNHG14 and up-regulated miR-211-3 had low proliferation and and colony-forming ability.

### Down-regulated SNHG14 or elevated miR-211-3p represses migration and invasion of BCa cells

**Fig. 6:**
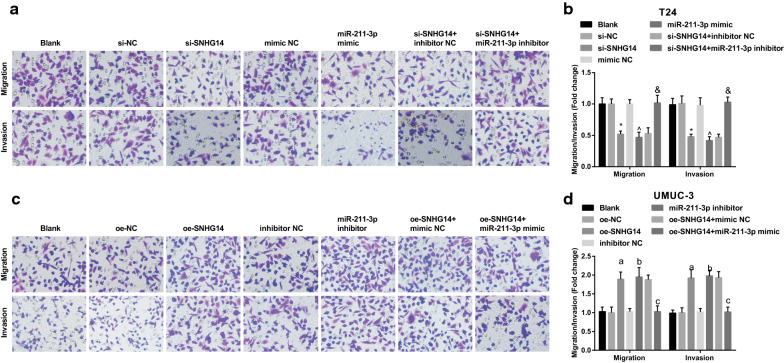
Decreased SNHG14 or elevated miR-211-3p depresses migration and invasion abilities of BCa cells. **a** The migration and invasion abilities of T24 cells after transfection tested via Transwell assay; **b** Quantification results of panel A. **c** The migration and invasion abilities of UMUC-3 cells after transfection tested via Transwell assay; **d** Quantification results of panel C; * vs the si-NC group, *P* < 0.05; ^ vs the mimic NC group, *P* < 0.05; and vs the si-SNHG14 + inhibitor NC group, *P* < 0.05; a vs the oe-NC group, *P* < 0.05; b vs the inhibitor NC group, *P* < 0.05; c vs the oe-SNHG14 + mimic NC group, *P* < 0.05. The experiment was repeated independently for 3 times, and the measurement data were expressed as mean ± standard deviation. One-way ANOVA was functioned for comparison among multiple groups, and Tukey’s post hoc test was used for pairwise comparison

Tumor cell migration is one of the most important characteristics of malignant tumors. Detecting tumor cell migration and invasion can indirectly reflect tumor migration or invasion ability, provide important information for exploring the mechanism of tumor occurrence and metastasis and indicate the biological functions of tumor cells. We tested the migration and invasion ability of cells after transfection by Transwell assay. The results revealed that in T24 cells, versus the si-NC and the mimic NC groups, the migration and invasion of cells in the si-SNHG14 and the miR-211-3p mimic groups were obviously decreased (all *P* < 0.05). Versus the si-SNHG14 + inhibitor NC group, the migration and invasion in the si-SNHG14 + miR-211-3p inhibitor group were distinctly strengthened (all *P *< 0.05) (Fig. 6a, b). With the si-SNHG14 + oe-NC group by contrast, the cell migration and invasion ability was enhanced in the si-SNHG14 + oe-ESM1 group (Additional file 1: Fig. 1I).

In UMUC-3 cells, versus the oe-NC and the inhibitor NC groups, the migration and invasion of cells were promoted in the oe-SNHG14 and the miR-211-3p inhibitor groups (all *P* < 0.05). In comparison with the oe-SNHG14 + mimic NC group, the migration and invasion of cells in the oe-SNHG14 + miR-211-3p mimic group were apparently impaired (both *P *< 0.05) (Fig. 6c, d). Versus the oe-SNHG14 + sh-NC group, weakened cell migration and invasion ability was detected in the oe-SNHG14 + sh-ESM1 group (Additional file 1: Fig. 1J). In summary, the cells with down-regulated SNHG14 and up-regulated miR-211-3 had low cell migration and invasion ability.

### Silenced SNHG14 or elevated miR-211-3p represses cell cycle entry and facilitates apoptosis of BCa cells

**Fig. 7:**
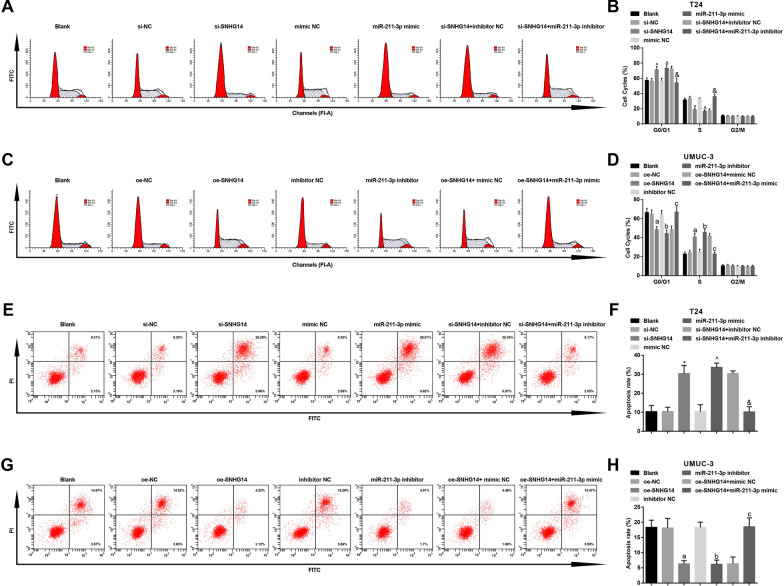
Decreased SNHG14 or elevated miR-211-3p inhibits cell cycle entry and facilitates apoptosis of BCa cells. **a** The cell cycle distribution of T24 cells after transfection tested via flow cytometry; **b** The proportion of T24 cells at G0/G1, S, G2/GM phases after transfection tested via flow cytometry; **c** The cell cycle distribution of UMUC-3 cells after transfection tested via flow cytometry; **d** The proportion of UMUC-3 cells at G0/G1, S, G2/GM phases after transfection tested via flow cytometry; **e** Apoptosis of T24 cells after transfection tested via flow cytometry; **f** Quantification results of panel E; **g** Apoptosis of UMUC-3 cells in each group after transfection tested via flow cytometry; **h** Quantification results of panel G; * vs the si-NC group, *P* < 0.05; ^ vs the mimic NC group, *P* < 0.05; and vs the si-SNHG14 + inhibitor NC group, *P* < 0.05; a vs the oe-NC group, *P* < 0.05; b vs the inhibitor NC group, *P* < 0.05; c vs the oe-SNHG14 + mimic NC group, *P* < 0.05. The experiment was repeated independently for 3 times, and the measurement data were expressed as mean ± standard deviation. One-way ANOVA was functioned for comparison among multiple groups, and Tukey’s post hoc test was used for pairwise comparison

Tumor is a kind of disease closely related to cell cycle. Analysis of cell cycle can observe the changes of tumor cell ploidy, which is helpful for early detection and treatment of tumor. To clarify the proportion of tumor cells in each phase is of great significance for drug development. Cell apoptosis exists in almost all tumor tissues and inducing apoptosis in tumor cells is also the key to treat tumors. In the study, we tested the cell cycle and apoptosis rate after transfection by PI single staining and AnnexinV-FITC/PI double staining. In T24 cells, in contrast with the si-NC and the mimic NC groups severally, the proportion of cells in G0/G1 phase was obviously elevated, the proportion of cells in S phase was apparently decreased, and the apoptosis rate was distinctly increased in the si-SNHG14 and the miR-211-3p mimic groups (all *P* < 0.05). In comparison with the si-SNHG14 + inhibitor NC group, the proportion of cells in G0/G1 phase was obviously reduced, the proportion of cells in S phase was clearly elevated, and the apoptosis rate was distinctly decreased in the si-SNHG14 + miR-211-3p inhibitor group (all *P *< 0.05) (Fig. 7a, b, e, f). Versus the si-SNHG14 + oe-NC group, the si-SNHG14 + oe-ESM1 group had a lower proportion of cells in G0/G1 phase, a higher proportion of cells in S phase, and a lower apoptosis rate (Additional file 1: Fig. 1k, m).

In UMUC-3 cells, with respect to the oe-NC and the inhibitor NC groups severally, the proportion of cells in G0/G1 phase was obviously reduced, the proportion of cells in S phase was clearly elevated, and the apoptosis rate was distinctly decreased in the oe-SNHG14 and the miR-211-3p inhibitor groups (all *P* < 0.05). In contrast with the oe-SNHG14 + mimic NC group, the proportion of cells in G0/G1 phase was distinctly elevated, the proportion of cells in S phase was clearly reduced, and the apoptosis rate was distinctly elevated in the oe-SNHG14 + miR-211-3p mimic group (all *P *< 0.05) (Fig. 7c, d, g, h). By comparison with the oe-SNHG14 + sh-NC group, the oe-SNHG14 + sh-ESM1 group had a higher proportion of cells in G0/G1 phase, a lower proportion of cells in S phase, and a higher rate of apoptosis (Additional file 1: Fig. 1l, n). All in all, knocking down SNHG14 and elevating miR-211-3p increased G0/G1 phase cells, inhibited cell malignant proliferation and promoted cell apoptosis in BCa.

### Down-regulated SNHG14 or elevated miR-211-3p reduces the tumor volume and weight of nude mice with BCa

**Fig. 8:**
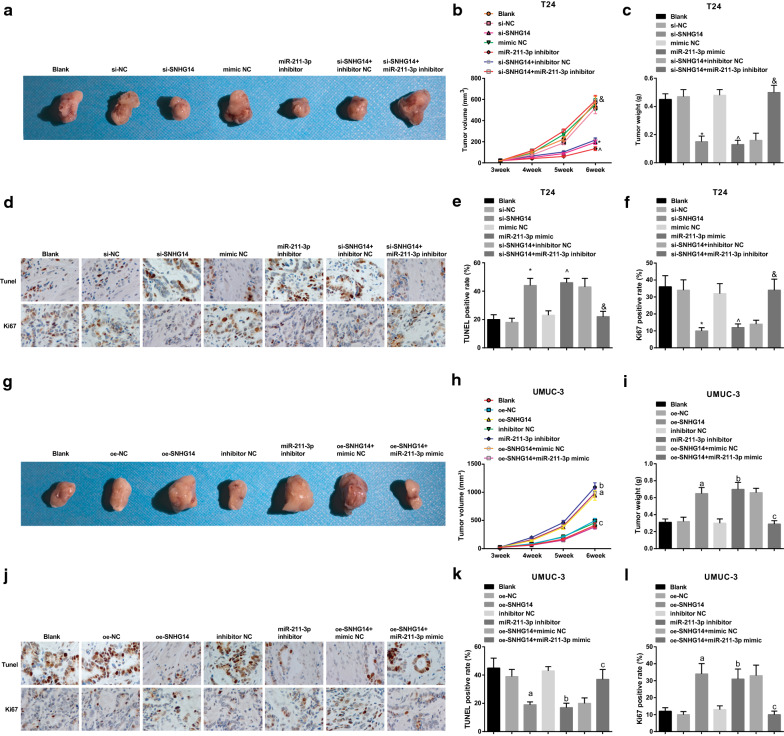
Silenced SNHG14 or elevated miR-211-3p reduces the tumor volume and weight of nude mice with BCa. **a** The xenografted tumor in nude mice transfected with T24 cells; **b** The tumor volume in nude mice after transfection with T24 cells; **c** The tumor weight of nude mice transfected with T24 cells; **d** TUNEL staining and Ki67 staining of tumor tissue sections in nude mice after transfection with T24 cells; **e** Cell apoptosis in nude mice after transfection with T24 cells detected by TUNEL staining; **f** Positive rate of Ki67 in nude mice after transfection with T24 cells detected by immunohistochemical staining; **g** The xenografted tumor in nude mice transfected with UMUC-3 cells; H. The tumor volume in nude mice after transfection with UMUC-3 cells; **i** The tumor weight of nude mice transfected with UMUC-3 cells; **j** TUNEL staining and Ki67 staining of tumor tissue sections in nude mice after transfection with UMUC-3 cells; **k** Cell apoptosis in nude mice after transfection with UMUC-3 cells detected by TUNEL staining; **l** Positive rate of Ki67 in nude mice after transfection with UMUC-3 cells detected by immunohistochemical staining; * vs the si-NC group, *P* < 0.05; ^ vs the mimic NC group, *P* < 0.05; and vs the si-SNHG14 + inhibitor NC group, *P* < 0.05; a vs the oe-NC group, *P* < 0.05; b vs the inhibitor NC group, *P* < 0.05; c vs the oe-SNHG14 + mimic NC group, *P* < 0.05. The experiment was repeated independently for 3 times, and the measurement data were expressed as mean ± standard deviation. One-way ANOVA was functioned for comparison among multiple groups, and Tukey’s post hoc test was used for pairwise comparison

Tumor xenograft in nude mice was applied to observe the growth of BCa tumors. Tumor growth was observed at 3rd week after injection. In T24 cells, versus the si-NC and the mimic NC groups severally, the tumor volume and weight in the si-SNHG14 and the miR-211-3p mimic groups were obviously decreased on the 4th week (all *P* < 0.05). In comparison with the si-SNHG14 + inhibitor NC group, the tumor volume and weight in the si-SNHG14 + miR-211-3p inhibitor group was distinctly elevated (all *P *< 0.05) (Fig. 8a–c). Then the tumor tissue sections were treated with TUNEL staining and Ki67 immunohistochemical staining (Fig. 8d–f). The findings turned out that tumor apoptosis was enhanced while the tumor proliferation was repressed in the si-SNHG14 and miR-211-3p mimic groups versus the si-NC group and mimic NC groups (all *P* < 0.05). The reduced apoptosis and enhanced proliferation presented in the si-SNHG14 + miR-211-3p inhibitor group versus the si-SNHG14 + inhibitor NC group (both *P* < 0.05).

In UMUC-3 cells, in contrast with the oe-NC, the inhibitor NC groups severally, the tumor volume and weight of nude mice were overtly increased in the oe-SNHG14 and the miR-211-3p inhibitor groups (all *P* < 0.05). In comparison with the oe-SNHG14 + mimic NC group, the tumor volume and weight of nude mice in the oe-SNHG14 + miR-211-3p mimic group were apparently reduced (all *P *< 0.05) (Fig. 8g–i). Moreover, versus the oe-NC and inhibitor NC groups, the tumor apoptosis was depressed whereas the proliferation ability was enhanced in the oe-SNHG14 group and miR-211-3p inhibitor groups (all *P* < 0.05). With the oe-SNHG14 + mimic NC group by contrast, the mice in the oe-SNHG14 + miR-211-3p mimic group manifested increased tumor apoptosis and decreased apoptosis (both *P* < 0.05) (Fig. 8j–l). It showed that down-regulating SNHG14 and up-regulating miR-211-3p had anti-tumor effects on BCa.

## Discussion

BCa is a familiar urological malignancy and one of the most expensive cancers to deal with around the world [21]. During the development and progression of BCa, plenty of tumor suppressors and oncogenes, consisting of certain miRNAs and lncRNAs, have been determined to be deregulated. For example, a recent study has indicated that SNHG14 participates in promoting the development and progression of BCa [9]. In addition, several article have revealed that some miRNAs, such as miR‑125b‑5p, miR-139-3p, miR-133a, and miR-142-3p, are implicated in the progression of BCa [1, 22]. In this study, we aimed to investigate the effect of SNHG14/miR-211-3p/ESM1 axis on the biological characteristics of BCa cells and eventually unveiled that SNHG14 negatively mediated miR-211-3p to enhance ESM1 expression, thereby promoting the malignant phenotype of BCa ((Additional file 2: Fig. S2).

The major finding of this work showed that up-regulated SNHG14 existed in BCa. The most obvious finding to emerge from the analysis was that decreased SNHG14 inhibited proliferation, migration, cell cycle entry and colony formation, and invasion abilities, and facilitated apoptosis of BCa cells, along with reduced the tumor volume and weight of nude mice with BCa. Consistently, SNHG14 has been found to be over-expressed in BCa tissues and cell lines [9]. Meanwhile, an article has indicated that the up-regulated SNHG14 could facilitate cell proliferation and accelerate cell cycle progression [23]. There is strong evidence that SNHG15 silence apparently depresses the invasion and epithelial-mesenchymal transition and proliferation, migration, while promotes apoptosis of breast cancer cells [24]. Of interest, SNHG14 up-regulation in clear cell renal cell carcinoma cells could stimulate cell to migrate and invade [25]. Other than that, it is known that SNHG14 depletion strains gastric cancer cell viability, migration, invasion, and contributes to enhanced apoptosis [26]. All in all, knocking down SNHG14 is an approach to hinder malignant phenotype of cancer cells. SNHG15 has a binding relationship with miR-211-3p in NSCLC [27]. However, the targeting relation between SNHG14 and miR-211-3p was still not confirmed previously.

The observation of this study was that inhibited miR-211-3p was presented in BCa. Another important finding was that up-regulated miR-211-3p restrained BCa cell progression, together with reducing tumor growth with BCa. The same expression trend of miR-211-3p is demonstrated in cancers. For instance, a article has revealed that miR-211-3p expression in non-small cell lung cancer (NSCLC) tissues is clearly decreased and over-expressed miR-211-3p could strain the migration and proliferation of A549 and H358 cells of NSCLC [27]. A study has verified that miR-211-5p excessive expression could accelerate the suppression in proliferation, invasion, and migration of papillary thyroid cancer [28]. Besides, miR-211-3p expression has been measured to suppress in colorectal cancer and lncRNA-uc002kmd.1-regulated miR-211-3p up-regulation could restrain cancer cell proliferation [12]. Similarly, in breast cancer, miR-211 expression is suggested to down-regulate and suppression of miR-211 facilitates lncRNA NEAT1 promoting cancer cell growth and invasion [29]. The suppressed miR-211 level is displayed in epithelial ovarian cancer tissues and miR-211 itself could arrest G0/G1-phase, limit proliferation and enhance apoptosis [30]. From all of those researches, miR-211-3p is further confirmed as the tumor suppressor.

The results of this study also revealed that up-regulated ESM1 was found in BCa which was regulated by miR-211-3p. ESM-1 is greatly elevated in clear cell renal cell carcinoma [31]. In the meantime, The Cancer Genome Atlas bioinformatics database analysis has suggested that ESM-1 expression is apparently increased in oral squamous cell carcinoma patients in contrast with that in normal individuals [32]. As to the targeting relation between miR-211-3p and ESM-1, Chen et al. pronounce that the possible participation of miRNAs in the pathogenesis of degenerative mitral valve disease with the in silico predicted target sites on plenty of genes, containing ESM1 [33].

## Conclusion

All in all, we conclude that silenced SNHG14 or elevated miR-211-3p depresses the proliferation, migration and invasion, and the tumor growth of nude mice, while facilitates the apoptosis of BCa cells, which may be connected with the down-regulated ESM1 expression. These findings may help us to understand BCa more deeply. However, the present study was conducted in a relative small scale and the related signaling pathway in SNHG14-mediated BCa shall be developed in the future studies. Additionally, more researches should be done for gaining insight into the molecular mechanisms of BCa in order to develop novel biomarkers and therapeutic targets for its diagnosis and treatment.

## Supplementary information

**Additional file 1: Figure S1.** ESM1 mediates SNHG14 to affect the development of BCa cells. **a** ESM1 expression in T24 cells was tested via RT-qPCR; **b** ESM1 expression in T24 cells was tested via Western blot assay; **c** ESM1 expression in UMUC-3 cells was tested via RT-qPCR; **d** ESM1 expression in UMUC-3 cells was tested via Western blot analysis; **e** The proliferation of T24 cells was tested via MTT assay; **f** The proliferation of UMUC-3 cells after transfection was tested via MTT assay; **g** The colony formation ability of T24 cells in each group was detected via colony formation assay; **h** The colony formation ability of UMUC-3 cells after transfection was detected via colony formation assay; **i** The migration and invasion abilities of T24 cells after transfection were tested via Transwell assay; **j** The migration and invasion abilities of UMUC-3 cells after transfection were tested via Transwell assay; **k-n** The cell cycle distribution and apoptosis of T24 and UMUC-3 cells after transfection were tested via flow cytometry; + vs the si-SNHG14 + oe-NC group, *P* < 0.05; **d** vs the oe-SNHG14 + sh-NC group, *P* < 0.05; The experiment was repeated independently for 3 times, and the measurement data were expressed as mean ± standard deviation. One-way ANOVA was functioned for comparison among multiple groups, and Tukey’s post hoc test was used for pairwise comparison.

**Additional file 2: Figure S2.** Experimental mechanism histogram. SNHG14 mediates miR-211-3p to target ESM, thereby promoting the malignant phenotype of BCa. SNHG14 and ESM1 are up-regulated, and miR-211-3p is down-regulated in BCa.

## Data Availability

Not applicable.

## Abbreviations

BCa: Bladder cancer
lncRNA: Long noncoding RNA
SNHG14: Small nucleolar RNA host gene 14
miRNAs: MicroRNAs
ESM1: Endothelial cell-specific molecule 1
TNM: Tumor node metastasis
RT-qPCR: Reverse transcription quantitative polymerase chain reaction
NC: Negative control
GAPDH: Glyceraldehyde-3-phosphate dehydrogenase
WT: Wild type
MUT: Mutant type
OD: Optical density
PI: Propidium iodide
ANOVA: One-way analysis of variance
NSCLC: Non-small cell lung cancer
