# Supplementary figures and images for: Silenced lncRNA SNHG14 restrains the biological behaviors of bladder cancer cells via regulating microRNA-211-3p/ESM1 axis
Source: Cancer Cell Int. 2021 Jan 22;21:67. doi: 10.1186/s12935-020-01717-7 (PMC7821404; doi:10.1186/s12935-020-01717-7)

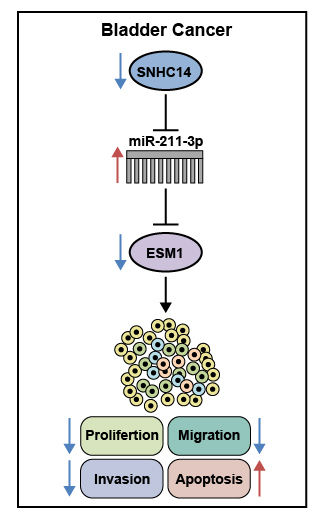

Supplement: Supplementary file 2 — Additional file 2: Figure S2. Experimental mechanism histogram. SNHG14 mediates miR-211-3p to target ESM, thereby promoting the malignant phenotype of BCa. SNHG14 and ESM1 are up-regulated, and miR-211-3p is down-regulated in BCa. [file 12935_2020_1717_MOESM2_ESM.jpg]
